# Personal News Items

**Published:** 1914-09

**Authors:** 


### Personal News Items.

Dr. Willis Moore is now permanently located at Hamilton, Texas.

Dr. W. C. Kimbrough, of Denton, has been quite ill for some time past.

Dr. I. J. Morris, of Dallas, has gone to New York and Boston to take a clinical course.

Dr. Joe E. Dildy and family, of Lampasas, have been enjoying the sea breezes at Galveston.

Dr. R. W. Baird and family, of Dallas, have returned from an extended trip through Canada.

Dr. W. O. Wilkes, of Waco, is in the East doing research work. Mrs. Wilkes accompanied him.

On August 13th a message was received from Dr. and Mrs. J. E. Gilcreest stating that they were sailing that day for New York.

Drs. C. P. Yeager and F. U. Painter, of Corpus Christi, have been in the East doing post-graduate work in surgery and dentistry.

Dr. E. H. Cary and Dr. G. M. Hackle, of Dallas, were among the Texans caught in Europe at the beginning of the war. It is needless to say they beat a hasty retreat.

Dr. F. P. Herff, of San Antonio, is planning in the near future to build a $20,000 home.

Dr. E. J. Nethery, of Sherman, has just returned from a ten-days Mayo clinic in Rochester, Minn.

Dr. G. H. Sandefer has gone to Denver, Colorado, where he will take a six months' course in medicine.

Dr. J. H. Hendrix, of Edgewood, has recently undergone an operation for gall stone. He is improving nicely and will soon be out again.

Dr. Killough, of Amarillo, has been doing post-graduate work in Vienna this summer. The doctor is expected home about the first of November, but his friends are hoping that he will change his plans and come sooner.

Dr. J. M. Horn, of Brownwood, and Dr. O. N. Mayo, of Temple, have just returned from a trip made for special surgical and medical study in the North and East.

Dr. F. May McAdams, of Galveston, has recently taken charge of the Department of Pathology in the Bryan Hospital. Dr. McAdams is a graduate of the medical branch of the State University at Galveston, and has been teaching in that institution since she graduated.

